# Lateral diffusion of rhodopsin in photoreceptor membrane: a reappraisal

**Published:** 2009-08-28

**Authors:** Victor I. Govardovskii, Darya A. Korenyak, Sergei A. Shukolyukov, Lidia V. Zueva

**Affiliations:** Institute for Evolutionary Physiology and Biochemistry, Russian Academy of Sciences, St. Petersburg, Russia

## Abstract

**Purpose:**

In a series of works between 1972 and 1984, it was established that rhodopsin undergoes rotational and lateral Brownian motion in the plane of photoreceptor membrane. The concept of free movement of proteins of phototransduction cascade is an essential principle of the present scheme of vertebrate phototransduction. This has recently been challenged by findings that show that in certain conditions rhodopsin in the membrane may be dimeric and form extended areas of paracrystalline organization. Such organization seems incompatible with earlier data on free rhodopsin diffusion. Thus we decided to reinvestigate lateral diffusion of rhodopsin and products of its photolysis in photoreceptor membrane specifically looking for indications of possible oligomeric organization.

**Methods:**

Diffusion exchange by rhodopsin and its photoproducts between bleached and unbleached halves of rod outer segment was traced using high-speed dichroic microspectrophotometer. Measurements were conducted on amphibian (frog, toad, and salamander) and gecko rods.

**Results:**

We found that the curves that are supposed to reflect the process of diffusion equilibration of rhodopsin in nonuniformly bleached outer segment largely show production of long-lived bleaching intermediate, metarhodopsin III (Meta III). After experimental elimination of Meta III contribution, we observed rhodopsin equilibration time constant was threefold to tenfold longer than estimated previously. However, after proper correction for the geometry of rod discs, it translates into generally accepted value of diffusion constant of approximately 5×10^−9^ cm^2^ s^−1^. Yet, we found that there exists an immobile rhodopsin fraction whose size can vary from virtually zero to 100%, depending on poorly defined factors. Controls suggest that the formation of the immobile fraction is not due to fragmentation of rod outer segment discs but supposedly reflects oligomerization of rhodopsin.

**Conclusions:**

Implications of the new findings for the present model of phototransduction are discussed. We hypothesize that formation of paracrystalline areas, if controlled physiologically, could be an extra mechanism of cascade regulation.

## Introduction

The present scheme of phototransduction in vertebrate photoreceptors rests on the assumption of free movement of components of signaling cascade within photoreceptor membrane. The molecule of visual pigment rhodopsin (Rh), upon absorption of light, converts into its enzymatically active form, metarhodopsin II (Meta II). Meta II, in the process of Brownian motion within the membrane, collides with specific photoreceptor G-protein transducin (T) and catalyzes exchange of GDP for GTP on its α-subunit. Charged with GTP, active T*_α_*GTP then meets and binds to cGMP-phosphodiesterase (PDE) thus greatly enhancing its catalytic activity toward cGMP. Cytoplasmic cGMP level decreases, and this leads to closure of cGMP-gated ionic channels in plasma membrane thereby generating electrical response. Due to free mobility of proteins in photoreceptor membrane, single Meta II collides with roughly 20,000 transducins per second, and activates approximately 200 of them. This multiplication comprises the first step of amplification in phototransduction. Turn-off of the cascade, i.e., its return to “dark” state, also relies on diffusion encounter between proteins. Active Meta II is quenched by its phosphorylation with rhodopsin kinase and by subsequent capping with arrestin. Active T/PDE complex is timely shut off by RGS9-Gβ5+R9AP (regulator of G-protein signaling and its membrane anchor protein).

The molecular ratio Rh: T: PDE in dark-adapted rod outer segment (ROS) is approximately 300: 25: 1. Therefore, prearranged Rh/T and T/PDE complexes, if they existed, could not significantly contribute to overall function of the cascade. The same is true for turn-off stages. Obviously, free mobility of phototransduction proteins, which are mostly membrane-bound, is a prerequisite condition for the phototransduction cascade to work (for reviews, see [1-6]).

Lateral diffusion of rhodopsin in photoreceptor membrane has been demonstrated in a series of works dating between 1973 and 1990 [7-15]. Mostly, the approach was to measure exchange by rhodopsin molecules between two halves of the same outer segment after bleaching one of the halves. Under microspectrophotometer, two narrow beams of light were passed symmetrically at two sides of the ROS, and concentration of (unbleached) rhodopsin was monitored by recording absorbance changes within the main α−band of the rhodopsin spectrum. It appeared that absorbance difference between the two sides of ROS produced by a bleaching flash further dissipates in a few tens of seconds, supposedly reflecting diffusion equilibration within the ROS discs. Only lateral movement across the ROS axis, i.e., in the disc plane, was detected; no diffusion along the ROS, i.e., between discs, occurred. Though there was some discrepancy between the numbers obtained by different groups, a consensus has been achieved. It is accepted that the diffusion constant for rhodopsin is roughly 5×10^−9^ cm^2^ s^−1^, or 0.5 μm^2^ s^−1^. This allowed estimating the viscosity of disc membrane that appeared to be 2 Poise. The value agreed well with the viscosity determined from measurements of fluorescence photobleaching and recovery [15], and from rotational diffusion coefficient of rhodopsin measured with absorbance spectroscopy [16,17].

However, it has recently been suggested that rhodopsin in the photoreceptor membrane exists in a dimeric form, and that dimers may form extended areas of paracrystalline organization [18-20]. Such organization seems incompatible with earlier data on free rhodopsin diffusion. Moreover, if permanently present in physiologic conditions, paracrystalline arrays of rhodopsin dimers would completely stop phototransduction. However, dynamic control of formation of paracrystalline areas could be an additional mechanism of cascade regulation. Therefore, we decided to re-investigate lateral diffusion of rhodopsin with more modern techniques not available in 1970s. Our fast-scanning dichroic microspectrophotometer allowed us to monitor entire spectral changes with sufficient time resolution, and to discriminate between rhodopsin and products of its photolysis [21,22]. So we used it to trace the lateral diffusion of rhodopsin and its photoproducts in the rod disc membrane of a few amphibian species and in gecko.

We found that absorbance changes within rhodopsin α−band after bleaching may result not only from rhodopsin diffusion but also from generation and decay of late bleaching products, Meta I and Meta III [22-25]. Meta III formation can simulate absorbance changes attributed earlier to diffusion of rhodopsin from unbleached to bleached part of the outer segment. We developed experimental approaches to eliminate the artifact and measured the diffusion constant for rhodopsin lateral mobility that appeared to be threefold to fivefold lower than thought before. We also confirmed the previous data [12] on presence of substantial immobile rhodopsin fraction within ROS that could be tentatively attributed to formation of rhodopsin paracrystalline areas.

## Methods

### Experimental animals

Experiments were conducted on retinal rods of salamander (*Ambystoma mexicanum*), frog (*Rana temporaria*), toad (*Bufo bufo*), and gecko (*Gecko gecko*). Adult (2 to 3 year-old) frogs and toads were captured in wild in St. Petersburg neighborhood. Six- to twelve-month old salamanders and geckos were obtained from a local breeder. Animals were treated in accordance with the Council for International Organizations of Medical Sciences (CIOMS) principles for biomedical research involving animals (1985). Frogs were kept in water tanks (3 l water per 20 frogs) at 4 °C. In these conditions frogs were hibernating and could be kept unfed for at least six months. Animals were used in experiments during first four months. Toads lived at room temperature in basins. They were given free access to running water, and fed live crickets and mealworms. Salamanders were kept in aerated aquariums (30 l tank per 3 to 4 animals) at 18 to 20 °C and fed minced meat and mosquito larvae. Both salamanders and toads were kept on a natural day-night light cycle.

Geckos lived in a terrarium at 25 °C and were fed live crickets and young cockroaches. The terrarium was dimly illuminated with heating red lamps, and a dark shelter was provided for the geckos.

Considering the source of food: there is no commercial supply of crickets, cockroaches, and meal worms in St. Petersburg. The insects and fly larvae (meal worms) were obtained from the Institute animal facility.

Prior to the experiment, animals were dark-adapted overnight at room temperature. Animals were double-pithed and decapitated, and the eyes removed under dim red light. Further dissection was made under infrared surveillance.

### Solutions

Physiologic saline used for retina isolation, storage and preparation of microspectrophotometer (MSP) samples contained for amphibians: 110 mM NaCl, 2.5 mM KCl, 1 mM CaCl_2_, 1 mM MgSO_4_, and 10 mM glucose. The solution was buffered to pH 7.5 with 10 mM Na-HEPES. This solution will further be referred to as standard Ringer. In experiments at acidic pH, HEPES was replaced with 10 mM Na-phosphate buffer, pH 6.3. In experiments with hydroxylamine, freshly neutralized hydroxylamine was added to standard Ringer at pH 7.5 to a final concentration of 50 mM. For geckos, 40 mM NaCl was added to standard Ringer to increase osmolarity. Additionally, the concentration of hydroxylamine, if present, was reduced to 10 mM. The gecko cone-like visual pigment was stable in this condition while metaproducts were quickly converted to retinaloxime. Glucose was purchased from ACROS (Morris Plains, NJ); all other chemicals were from Sigma-Aldrich Chemie (Saint Louis, MO). All measurements were done at a temperature between 20 and 22 °C.

### Preparation of MSP samples

To make MSP samples, we immersed the eye into standard Ringer, opened the eye along the equator, hemisected the eye cup, and detached retinal pieces from underlying pigment epithelium. Samples could be used immediately or stored in a light-tight chamber at 4 °C up to one day. Measurements were made either on “sealed” or perfused samples. To prepare a sealed sample, small retinal pieces were shaken in a drop of appropriate saline on coverslip to obtain isolated rods and ROSs. Then the sample was covered by another coverslip and sealed at the edges with petroleum jelly. If measurements at acidic pH or in the presence of hydroxylamine were intended, retinal pieces were incubated for 10 min in the corresponding solution and sealed in the same solution. For recording from (partially) intact cells perfused with standard Ringer, the retina was chopped with a razor blade and the pieces transferred to the perfusion chamber that was formed by two coverslips separated by a roughly 200 µ gap. Perfusion solution was gravity-fed into the chamber and sucked out at the outlet.

### Instrument

This is a laboratory-made instrument whose design is described in [21,22]. The MSP is a single-beam instrument where the photomultiplier output (Hamamatsu R1463 tube) is fed to the computer memory via a current-to-voltage transducer and a 16 bit A/D converter. A halogen lamp served as a light source both for visible and near-UV (down to 340 nm) region. An adjustable mask was placed at monochromator exit slit and imaged, with demagnification, in the sample plane. For demagnification, a quartz-mirror microscope objective (40×, N.A. 0.5) was used [22]. The instrument was supplied with infrared TV camera for sample viewing and adjustments.

A fast spectral scanning mechanism allowed recording complete absorbance spectrum of a single ROS between 340 and 800 nm in 500 ms. A polarizer in the measuring beam was under computer control. Thus, the absorbance spectra, when scanned from longer to shorter wavelengths and in the opposite direction, could be recorded at two mutually perpendicular directions of polarization: across (T, transverse) and along (L, longitudinal) the ROS axis. Two-polarization recording took about 1 s.

To adapt the instrument to diffusion measurements, we placed a glass plate between the beam mask and the condenser. The plate was attached to a computer-controlled wobbler, so the measuring beam could be switched alternatively between two sides of the ROS. The beams were placed symmetrically at one-fifth the ROS width from ROS edges. When recording complete absorbance spectra at the two positions, we used a nonattenuated beam intensity, with a result of 0.5% bleach per spectral scan. For continuously monitoring absorbances at a single wavelength, beam intensity was reduced 100 fold. In this regime bleaching did not exceed 2% during typical 300 s duration time scan. Width of the measuring beam was set to 2 μm for fat salamander rods, and 1.5 μm for more slender frog and toad rods.

Bleaching light was provided by high-intensity light-emitting diode (green 525 nm, #110104; Marl International Ltd., Ulveston, Cumbria, UK). An adjustable mask was placed into the bleaching beam to protect one side of the ROS from bleaching. Mutual positioning of the ROS, measuring and bleaching beams was controlled with infrared TV. Dimensions of all cells were determined from captured TV images.

## Results

### Earlier data on rhodopsin diffusion are distorted by Meta III formation

**Figure 1:**
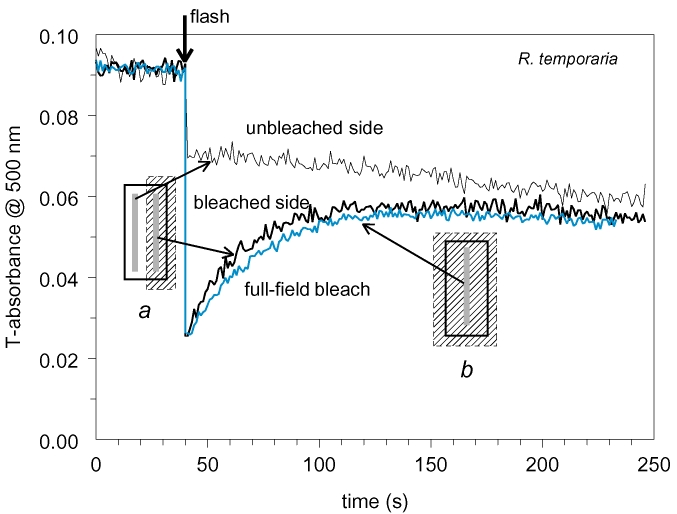
Possible artifact when measuring lateral diffusion of rhodopsin. The two black noisy lines are absorbance traces recorded in configuration *a*, with a measuring beam jumping alternatively between the two sides of ROS. The arrow indicates the moment at which a half-ROS bleaching flash was applied (hatched area in *a*). Difference between absorbances at two ROS sides generated by the flash further dissipates, supposedly due to diffusional exchange by rhodopsins. Bold blue line shows the time course of post-flash absorbance changes recorded in configuration *b*. Here the measuring beam is placed at the ROS center and full-field bleach applied, so no diffusion-related changes was expected. Recordings are made in standard Ringer solution at pH 7.5.

We started from repeating rhodopsin diffusion measurements accordingly to the protocol used in most of the previous works. Position of the measuring beam was alternatively switched between the two sides of the outer segment every 0.5 s and absorbance changes monitored at rhodopsin λ_max_ (Figure 1). During the initial period before the bleaching flash, placement of the measuring beams within the ROS was finely adjusted to achieve equal absorbances at both positions. Then the half-field bleaching flash was applied at 40 s (arrow) that resulted in a large imbalance between the illuminated (bleached) and (nominally) nonilluminated sides of the ROS. Further absorbances at the two positions started to converge, a process that has been previously attributed to the diffusion exchange by rhodopsin molecules within the ROS discs (black noisy lines in Figure 1). However, we found that if an immobile measuring beam was placed at the ROS axis and bleaching flash covered the entire ROS, post-bleach absorbance still rose along the curve closely approaching the curve registered with half-ROS bleach (blue line in Figure 1).

**Figure 2:**
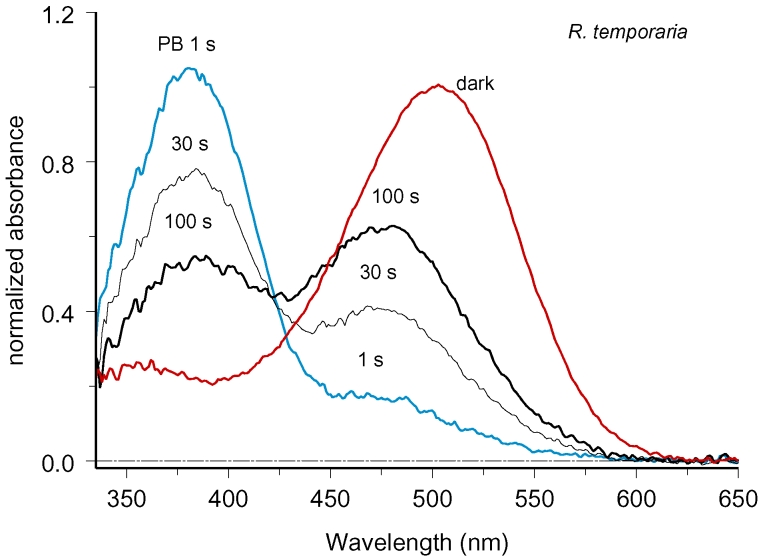
Metarhodopsin III formation can simulate rhodopsin diffusion. The series of spectra was recorded with the measuring beam placed at ROS center, in darkness and at various intervals after 1-s full-field bleach. The curves are color-coded to facilitate tracing individual spectra. Peak of Meta III at approximately 480 nm reaches its maximum at 100 s postbleach. Though λ_max_ of Meta III is blue-shifted compared to rhodopsin, Meta III spectrum has a long-wave tail that runs virtually parallel to the spectrum of rhodopsin. Therefore, there is no wavelength for diffusion measurements where Meta III contribution can be neglected. Recordings are made in standard Ringer solution at pH 7.5. Spectra represent average of seven cells.

Figure 2 explains the reason for this behavior. Here a series of spectra was recorded with centrally located beam in darkness and at various moments of time after full-field bleach. Immediately after the bleaching flash, high Meta II peak appeared at 380 nm. Meta II was in equilibrium with Meta I (subpeak at 475 nm). Then Meta I and Meta II decayed and partially converted into Meta III, resulting in growing maximum at 475–480 nm (curves recorded at 30 and 100 s; [23]). Thus Meta III production could closely imitate the postbleach absorbance increase attributed to rhodopsin diffusion. It has been suggested that contribution of metaproducts can be avoided by tracing absorbances at the long-wavelength branch of rhodopsin spectrum (say, at 550 nm for frog) where absorbance of short-wave shifted Meta III could supposedly be neglected [9]. However, Figure 2 shows that Meta III absorbance, albeit peaking at shorter wavelength, had an extended long-wave tail that ran virtually parallel to the rhodopsin spectrum. Therefore, there was no “safe” wavelength at which diffusion measurements could be conducted. The same result was obtained in all amphibian species studied.

### Eliminating contribution of metaproducts by measuring at acidic pH or in presence of hydroxylamine

**Figure 3:**
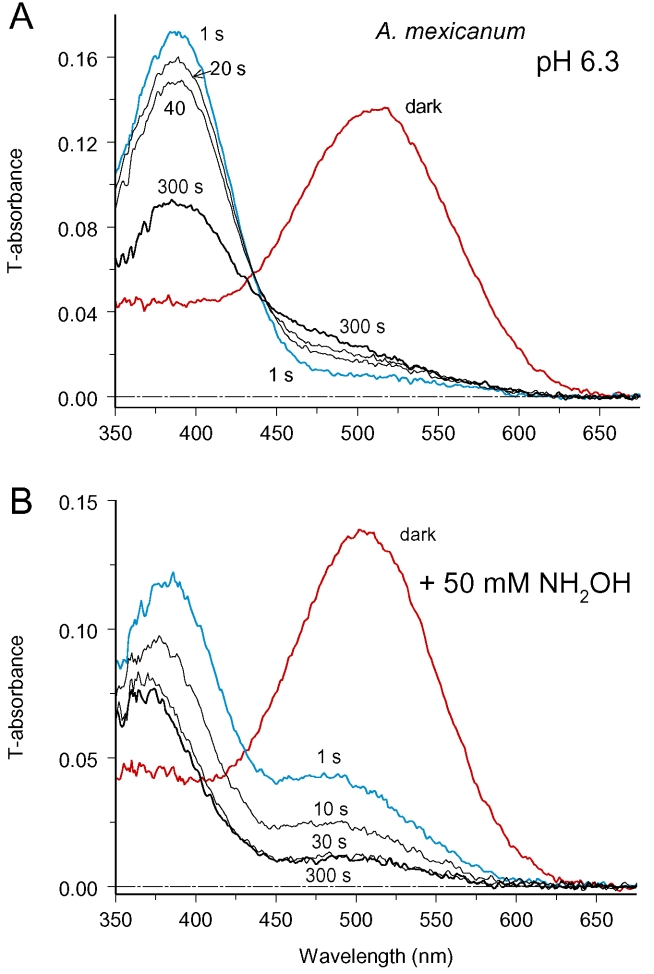
Two ways to eliminate the Meta III artifact. In **A**, dark and postbleach spectra were recorded at acidic pH. The amount of Meta III formed was greatly reduced, and its spectrum narrowed. Thus tracing rhodopsin diffusion at λ > 550 was only marginally compromised by Meta III formation. Spectra represent average of six cells. In **B**, recordings were made in standard Ringer at pH 7.5 with addition of 50 mM of freshly neutralized hydroxylamine. Conversion of metaproducts to retinaloxime was complete at 30 s postbleach, and afterwards metaproducts did not contribute to absorbance changes. Spectra represent average of five cells.

To eliminate the effect of metaproducts, we manipulated the ionic milieu of the sample. The first successful approach was to use acidic Ringer solution (pH 6.0 to 6.5). At acidic pH, the amount of Meta III was substantially reduced, and its absorbance spectrum narrowed (Figure 3A). Therefore, Meta III production would only marginally compromise measurements at λ≥560 nm in salamander or at λ≥550 nm in frog and toad rods.

The second approach was to use hydroxylamine, which quickly destroys metaproducts by converting retinal to retinaloxime. Retinaloxime (λ_max_=365 nm) did not perceptibly absorb within the main rhodopsin band so it did not interfere with diffusion measurements. It appeared that the effect of 50 mM hydroxylamine on metaproducts was virtually complete at 30 s postbleach (Figure 3B). Starting from this time, diffusion could be traced at visual pigment λ_max_.

### Postbleach equilibration is substantially slower than previously reported

**Figure 4:**
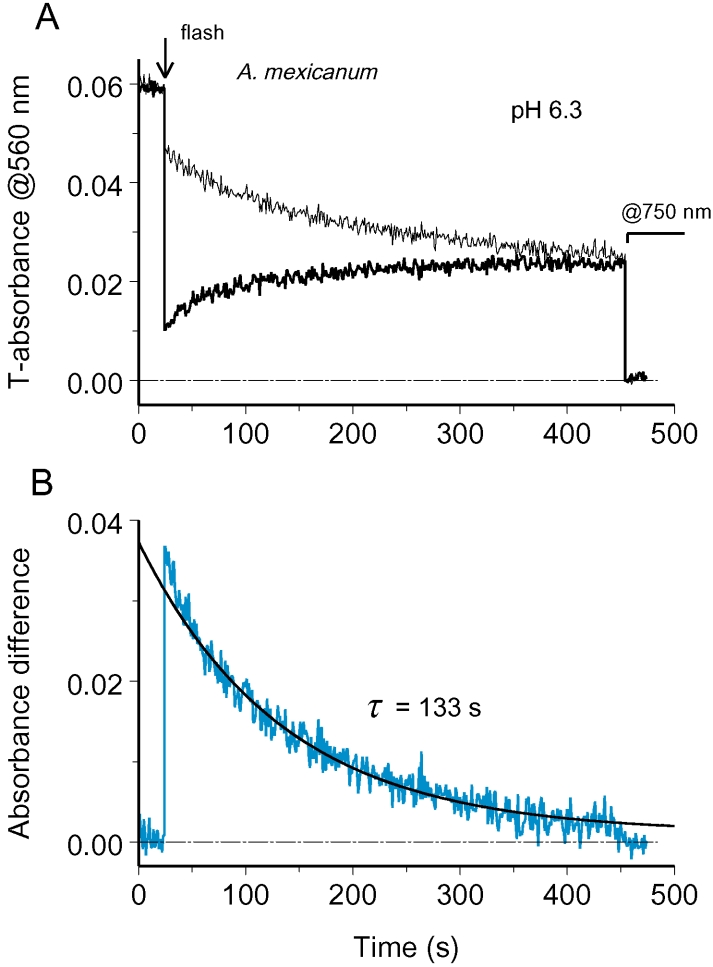
Tracing rhodopsin diffusion at acidic pH. **A:** Absorbance changes at two sides of a ROS after half-ROS bleach (configuration *a* in Figure 1) converge demonstrating diffusion exchange by rhodopsin. ROS diameter is 12.9 μ. Here and onward, thin black line shows absorbance changes at nominally unbleached side, while the black bold trace is recorded from the bleached side. Arrow marks the bleaching flash. The two traces were adjusted vertically to bring absorbances at 750 nm to zero (stretch starting from 450 s). **B:** Absorbance difference between unbleached and bleached side, shown by bold blue line, dissipated along an exponent with the time constant of 133 s (smooth black line). This yielded the apparent diffusion constant, *D_a_*=1.4×10^−9^ cm^2^s^−1^.

Figure 4A shows absorbance changes after half-field bleach of salamander ROS recorded in Ringer solution at pH 6.3. After tracing the absorbances at two sides of ROS for roughly 400 s post-bleach, the instrument was set to 750 nm where the visual pigment absorbance is negligible, and a 20-s stretch of zero absorbance was recorded. It further allowed aligning the curves with respect to zero line, thus excluding possible effects of light scattering and nonrhodopsin absorbance.

**Figure 5:**
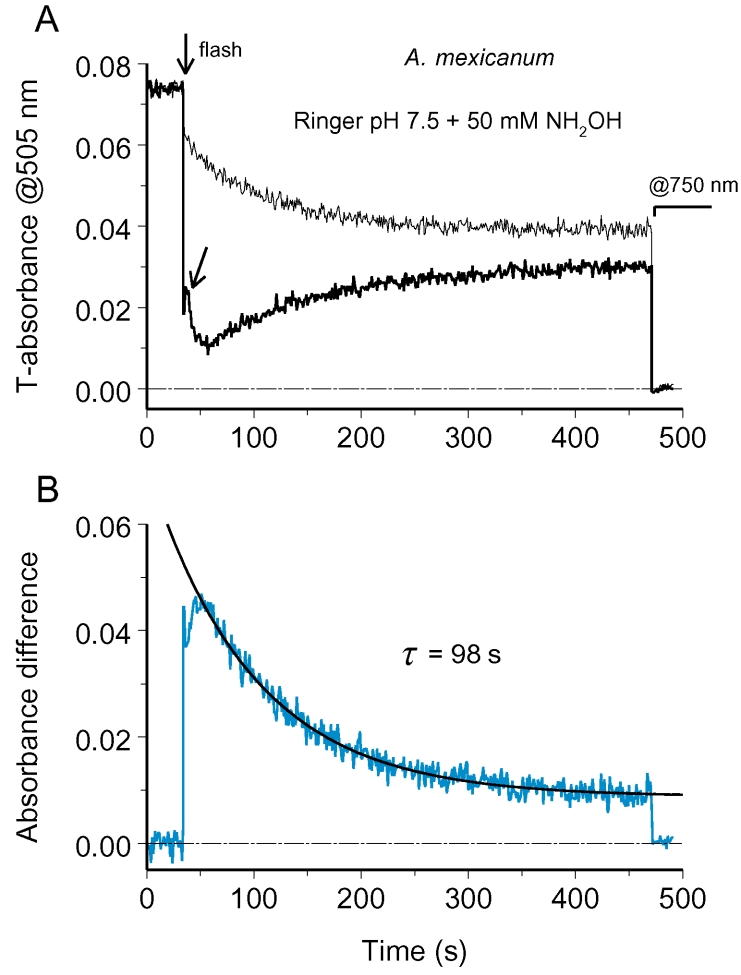
Tracing rhodopsin diffusion at physiologic pH in the presence of hydroxylamine. Oblique arrow in **A** points to initial absorbance increase at the bleached side due to the formation of extra Meta I-like product in hydroxylamine-containing solution. Multiphase absorbance difference changes seen up to 30 s postbleach in B were due to formation of metaproducts and their subsequent conversion to retinaloxime. ROS diameter is 11.6 μ, the apparent diffusion constant *D_a_*=1.3×10^−9^ cm^2^s^−1^.

After the initial steep stretch, rhodopsin concentration gradient dissipated along the exponential curve with the time constant of 133 s (Figure 4B). This is sixfold to tenfold slower than equilibration times reported previously for *Necturus* rods of approximately the same size (23 s [8]; 12 s [9]). Similar results were obtained at physiologic pH 7.5 in Ringer containing 50 mM hydroxylamine. As seen from Figure 5A (bold line), in the presence of NH_2_OH, absorbance changes at the bleached side of ROS showed a more complicated time course. Initial sudden drop produced by bleaching was followed by a brief transient increase (pointed by an oblique arrow) and then decrease that lasted for 25 s. After this the absorbance at the visual pigment λ_max_ steadily increased. Multiphase absorbance changes at the bleached side were caused by specific effects of hydroxylamine on metaproducts. As can be ascertained from recording complete absorbance spectra, NH_2_OH initially resulted in transient shift of Meta I – Meta II equilibrium toward Meta I or Meta I-like product, compared to the level immediately after bleaching [25]. Only after this, hydroxylamine converted metaproducts to retinaloxime. The conversion was complete at 30 s postbleach (Figure 3B), and after that, the absorbance difference truly reflected diffusion exchange by rhodopsin between the bleached and unbleached sides of the ROS (Figure 5B).

The equilibration time constant in this cell was a bit shorter than in the ROS shown in Figure 4 (98 s versus 133 s), which partly resulted from its smaller diameter *L* (11.6 μm versus 12.9 μm). If the apparent diffusion constant is calculated using the formula [8,9] as shown (Equation 1),

$${\text{D}}_{\text{a}}={\text{L}}^{\text{2}}/\left(\pi^{\text{2}}\tau \right)$$

the two cells yield close values (1.4×10^−9^ cm^2^s^−1^ and 1.3×10^−9^ cm^2^s^−1^, respectively). See, however, Discussion section on converting the rates of equilibration into *D* values.

### Substantial fraction of rhodopsin in ROS can be virtually immobile

**Figure 6:**
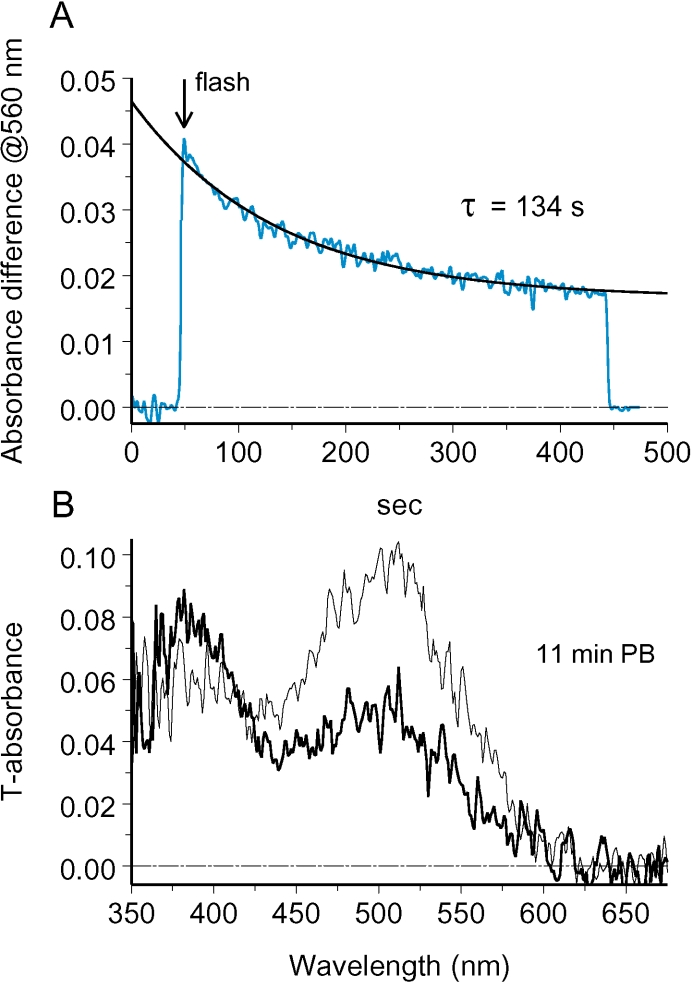
Equilibration of rhodopsin and its bleaching products can be incomplete. **A:** A shows absorbance difference between unbleached and bleached side after half-field bleach. **B**: Spectra at 11 min postbleach showed that an extra rhodopsin (peak at roughly 515 nm) remained in the unbleached ROS half while photoproducts (retinal and Meta II, peak at 380 nm) stayed predominantly in the bleached half. Nonequilibrating rhodopsin fraction comprised 41% of original concentration difference. Measurements are made on salamander ROS in Ringer pH 6.3.

As is already seen in Figure 5, the equilibration between two sides of ROS may be incomplete. A certain fraction of rhodopsin gradient created by bleaching may persist for a long time. A more prominent example of incomplete equilibration is shown in Figure 6A, where 42% of the gradient created by the bleaching flash persisted for tens of minutes. Spectra recorded at 11 min postbleach showed that a substantial fraction of rhodopsin stayed at the unbleached side while bleaching products (peak at 380 nm) remained in the bleached half of the ROS (Figure 6B). In a few cells, no equilibration was observed, so the immobile rhodopsin fraction could comprise 100% (data not shown).

**Figure 7:**
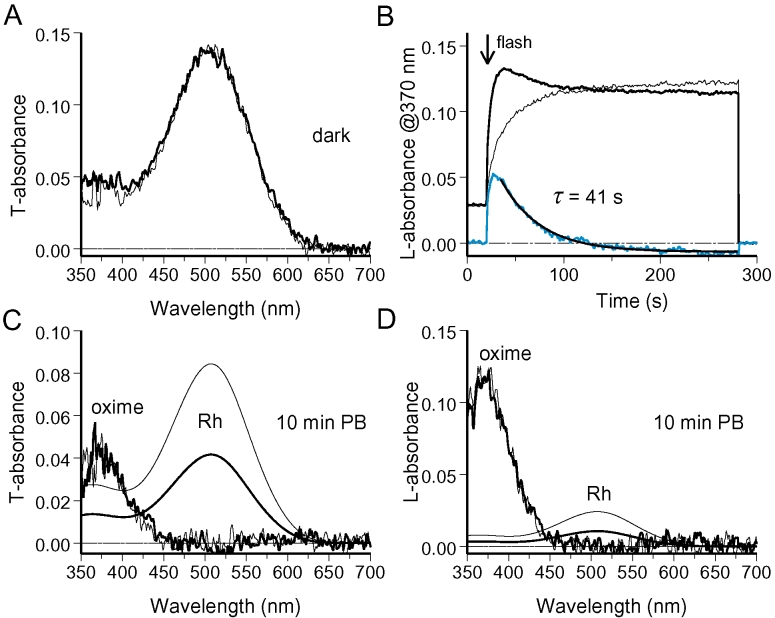
Steady rhodopsin gradient in amphibian ROSs persists while retinaloxime equilibrates quickly and completely. **A:** Initial dark absorbance spectra recorded at two sides of a salamander ROS in standard Ringer pH 7.5+50 mM hydroxylamine show even distribution of rhodopsin. **B:** Absorbance changes at bleached and unbleached sides of the ROS at 370 nm and L-polarization trace formation and subsequent equilibration of retinaloxime. **C, D** show T- and L-absorbance spectra at two sides of ROS after 10 min postbleach. Smooth lines are visual pigment template fits to long-wave peaks of postbleach absorbance spectra. They show grossly nonequilibrium distribution of rhodopsin. Noisy curves were obtained by subtracting template curves from the postbleach spectra. Retinaloxime peaks at approximately 370 nm were equilibrated, pointing to the structural continuity of the disc membrane.

Basically the same result was obtained in NH_2_OH-containing Ringer, with a notable feature. While a substantial rhodopsin gradient could persist for half an hour (maximum time tested), retinaloxime completely equilibrated in a couple of minutes. Figure 7 shows the result of such an experiment. Figure 7A shows dark-adapted absorbance spectra at two symmetrically located sites in the ROS. The absorbances at the two locations were continuously monitored at 370 nm (Figure 7B). This wavelength corresponded to λ_max_ of the oxime of all-*trans* retinal/3,4-dehydroretinal chromophore mixture present in the cell. Polarization of the measuring beam was set across the disc plane (L), accordingly to predominant orientation of retinaloxime absorbing dipole. A half-field bleaching flash applied at 20 s (arrow) produced an absorbance difference that continued to rise for a while, reflecting the conversion of metaproducts at the bleached side to retinaloxime. Then absorbance curves at the two locations converged and finally intersected at 120 s. Figure 7C,D explains the reason for the intersection: while retinaloxime equilibrated completely, immobile rhodopsin fraction added extra absorbance at 380 nm in the unbleached half. By fitting the rhodopsin template to the long-wave peak of final spectra we were able to estimate the contribution of rhodopsin (smooth lines in Figure 7C,D) and subtract this from final T- and L-spectra. The residual peaks at 370 nm showed that retinaloxime equilibrated completely (noisy curves in Figure 7C,D), in spite of persisting rhodopsin gradient. Retinaloxime equilibrated roughly threefold faster than the movable fraction of rhodopsin (compare time constants of equilibration in Figure 4, Figure 5, Figure 6, and Figure 7).

**Figure 8:**
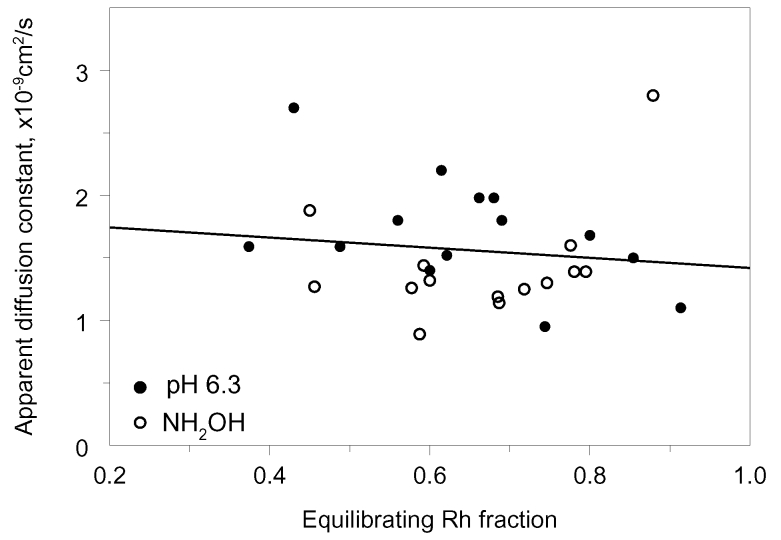
The apparent diffusion constant and the size of equilibrating rhodopsin fraction do not correlate. Data are obtained on 29 salamander ROSs, each point marks an individual cell. Filled circles represent recordings in Ringer pH 6.3. Empty circles indicate Ringer pH 7.5+50 mM hydroxylamine. Linear regression line drawn through pooled data has a slope of −0.4±0.65 (mean±SEM). Similar results were obtained on frog and toad rods.

All the data shown above, like in all previous works [7-15], were obtained on isolated rod outer segments lying on the bottom of sealed sample. This ensured good stability of ROS position with respect to measuring beams, but obviously conditions of the sample were far from physiologic. Since the immobile rhodopsin fraction varied greatly among cells, we tested whether the variability could in any way be related to the state of the cell. We conducted a series of measurements on salamander isolated rods that retained inner segment, nucleus, and sometimes the synaptic terminal, or on intact rods attached to small retinal pieces. The sample was perfused with a constantly flowing physiologic solution. In these samples, rods revealed their relatively “healthy” metabolic state by readily converting released retinal to retinol [26,27]. Retinol then equilibrated across the ROS quickly and completely, similarly to retinaloxime (data not shown). However, a substantial rhodopsin gradient persisted in many cells. We found no obvious difference in the size of immobile rhodopsin fraction between the isolated outer segments and intact cells. Also, we found no correlation between the mobile fraction and the diffusion constant in either of the amphibian species studied (Figure 8).

### Diffusion of visual pigment and retinaloxime in gecko photoreceptors

Quantitative interpretation of results on rhodopsin diffusion is complicated by the structure of ROS discs. The discs in amphibian rods are separated into numerous lobes by deep incisures that run from the disc's edge toward its center. The incisures create a petal-like structure that can substantially retard lateral diffusion of membrane components [8,9]. To avoid this complication, we tried recordings on rod-like photoreceptors of the Tokay gecko. It is reported that there are only a few (one or two) incisures in photoreceptors of nocturnal geckos [28]. An additional advantage is that the amount of Meta III in gecko is small even in standard Ringer (pH 7.5, no NH_2_OH treatment) [27], so the diffusion could be traced in less harsh environment.

**Figure 9:**
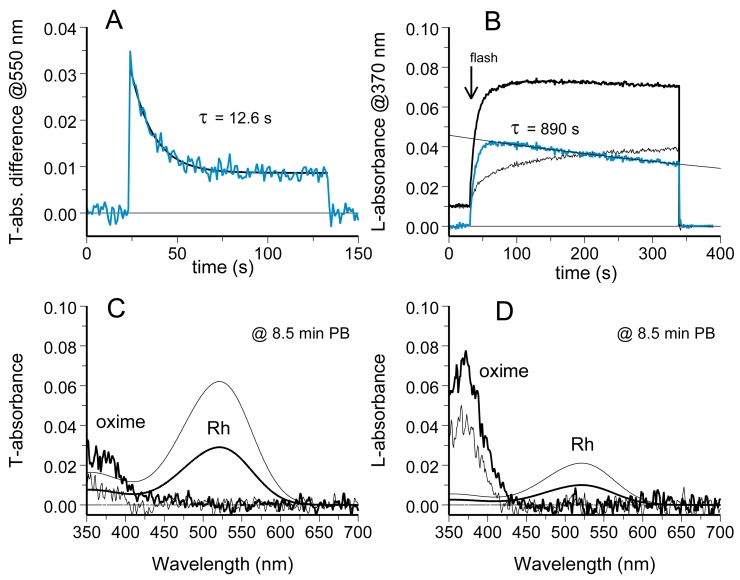
Diffusion of visual pigment and retinaloxime in gecko rods. **A** and **B-D** represent recordings from different cells. **A:** Dissipation of visual pigment concentration gradient after half-field bleach proceeds in gecko substantially faster than in amphibians, but is also grossly incomplete. **B:** Equilibration of retinaloxime in gecko ROS was more than by an order of magnitude slower than in frog or salamander. **C, D:** Absorbance spectra that were recorded at 8.5 min postbleach from the same cell as in **B**. Equilibration of both the visual pigment and retinaloxime was incomplete. Smooth lines are visual pigment template fits to the long-wave peaks of the postbleach absorbance spectra. Noisy curves were obtained by subtracting the template curves from the postbleach spectra and represent retinaloxime. Extra rhodopsin (peaks at approximately 520 nm) remained in the unbleached ROS half while retinaloxime (peaks at 365 nm) was formed and stayed predominantly in the bleached half.

Contrary to the expectation, it appeared that the results in gecko were more complicated than in salamander or frog. Similarly to amphibians, the extent of visual pigment equilibration in gecko varied greatly among cells, between virtually complete equilibration and total lack of diffusion. On average, immobile visual pigment fraction in gecko was approximately twice as big as in salamander (0.69 versus 0.34; see Table 1). This was due to a higher proportion of cells that exhibited virtually no diffusion (8 of 19 ROS, compared to 2 of 31 in salamander). Nevertheless, the diffusible fraction of visual pigment in gecko moved faster, with its equilibration time being threefold to fivefold shorter than in amphibian rods of similar diameter (Figure 9A). However, diffusion of retinaloxime could be greatly retarded in many cells (Figure 9B), so the final spectra at 10 min postbleach demonstrated rhodopsin mainly stayed at the unbleached side of the outer segment, and retinaloxime at the bleached side (Figure 9C, D). Possible explanation of the “aberrant” behavior of gecko photoreceptors will be considered in Discussion.

**Table 1:** Apparent diffusion constant and immobile rhodopsin fraction in different species.

**Species**	**Conditions**	***D_a_* (average±SEM) 10^−9^ cm^2^.s^−1^**	**Immobile Rh fraction (average±SEM)**
*Ambystoma mexicanum*			
	pH 6.3	1.44±0.12 (15)	0.35±0.04
	+NH_2_OH	1.7±0.11 (14)	0.33±0.03
*Rana temporaria*			
	pH 6.3	1.26±0.24 (7)	0.17±0.09
	+NH_2_OH	1.13±0.13 (8)	0.2±0.04
*Bufo bufo*			
	pH 6.3	0.71±0.12 (6)	0.33±0.04
	+NH_2_OH	0.55±0.11 (5)	0.29±0.09
*Gecko gecko*			
	pH 7.5	4.2±0.3 (11)	0.69±0.06 (19)

Statistics of apparent diffusion constant and immobile rhodopsin fraction in amphibians is presented separately for two conditions: at acidic pH and in the presence of hydroxylamine. There is no statistically significant difference between the results obtained in the two solutions. For gecko, data are obtained in standard Ringer at pH 7.5, no hydroxylamine. No correction for disks’ geometry is applied. Number of measured cells is given in parentheses.

The apparent visual pigment diffusion constants *D_a_* and the size of nonequilibrating rhodopsin fraction in rods of the four species studied are summarized in Table 1. The value of *D_a_* has been computed using Equation (1) that follows from the solution of diffusion equation after neglecting high-order terms ( [12]; see also Discussion). There was no statistically significant difference between values of *D_a_* or immobile rhodopsin fraction measured at acidic pH or in the presence of hydroxylamine, in either of the amphibian species studied (Table 1).

## Discussion

### Earlier data on rhodopsin diffusion are distorted by metaproduct formation

Our results suggest that appearance and decay of photolysis products may have significantly compromised earlier data. Spectral recordings show that absorbance increase at the bleached side of the ROS originates, at least partly, from formation of Meta III (Figure 1 and Figure 2). The problem passed apparently unnoticed by Poo and Cone [7,8], but was recognized by Liebman’s group. As a solution, Liebman et al. [9,11,12] traced the absorbance changes at a wavelength on the long-wave slope of rhodopsin spectrum rather than at the pigment’s *λ_max_*. However, Figure 2 shows that there is no “safe” wavelength that would exclude contribution of Meta III to the recordings at physiologic pH. At present, it is hard to tell what could be pH values in Poo & Cone [7,8] or Liebman et al. [9,11,12] samples that were tightly sealed between coverslips in the solution of unspecified composition. Yet our measurements, which were specifically designed to eliminate the effect of photoproducts, consistently yielded equilibration threefold to tenfold slower than reported in earlier works for rods of comparable diameter. The apparent diffusion constant derived from our data, 1.2×10^−9^ to 1.7×10^−9^ cm^2^s^−1^ (Table 1), is in good agreement with the estimate from the fluorescence photobleaching and recovery experiments that are insensitive to formation of metaproducts [15,29]. This, again, is threefold to fivefold lower than reported in earlier papers. Therefore we believe that previous value of diffusion constant following from absorbance measurements is an overestimate due to formation of Meta III that could mimic absorbance changes expected from rhodopsin diffusion (see next section).

### Estimating diffusion constant from equilibration time

To derive the value of diffusion constant from experimentally measured equilibration time course and ROS diameter, Poo & Cone [8] and Liebman et al. [9,12] represented the outer segment as a rectangular slab. There is a standard solution of diffusion equation for such a configuration that can easily be obtained from Equation 4.58 in [30]. It yields concentration profile of rhodopsin at any moment of time as a series of spatial cosine waves and time exponentials (Equation 2):

$$\Delta C(x,t)=\frac{\Delta C_{0}}{2}+2\cdot \Delta C_{0}\sum_{n=1}^{\infty }\frac{{\left(-1\right)}^{\frac{n-1}{2}}}{\pi n}\cos\left(\frac{\pi nx}{L}\right)\cdot \exp\left(-\frac{\pi^{2}n^{2}}{L^{2}}Dt\right)$$

Here Δ*C(x, t)* is the difference of concentrations between the two sides of ROS, Δ*C*_0_ is the initial step-wise concentration difference produced by the half-ROS bleach, *L* is the ROS diameter, *x* is the distance of measuring beam from the edge of the unbleached half, and *t* is time. Index *n* can only assume odd values. After a certain initial part that depends on the position of measuring beams within the ROS (*x*), high-order terms (n>1) can be neglected, and the time course of the dissipating concentration gradient follows a single exponential whose time constant is *τ=(L/π)^2^/D*. Solved for *D*, it yields Equation 1, which was used to calculate the values given in Table 1.

It should be noted that earlier authors used a somewhat different approach for determining *D* from recorded concentration time courses. Obviously due to the lack of curve-fitting software in the 1970s, Cone’s and Liebman’s groups [7-12,15] characterized dissipation of gradient with t_1/2_, time at which the gradient decreased to half of its value immediately after bleaching. Then Cone’s group [8] used simple relationship τ=*t*_1/2_/0.693 in Equation (1). Liebman and Entine [9] and Drzymala et al. [12] noticed that the accuracy of the result can be improved by using the formula

$$D=-\frac{L^{2}}{\pi^{2}t_{1/2}}\ln\left(\frac{-\pi /8}{\cos(\pi x/L)}\right)$$,

where *x* is the distance of measuring beam from the unbleached edge of ROS. Instead of relying on *t*_1/2_, we performed single-exponential fitting of the main part of dissipation curve. This corresponds to the first-order term of Equation 2 while higher-order terms are responsible for a small initial fast peak (Figure 4B, Figure 6A). Such a fit is grossly insensitive to the position of measuring beams in ROS.

**Figure 10:**
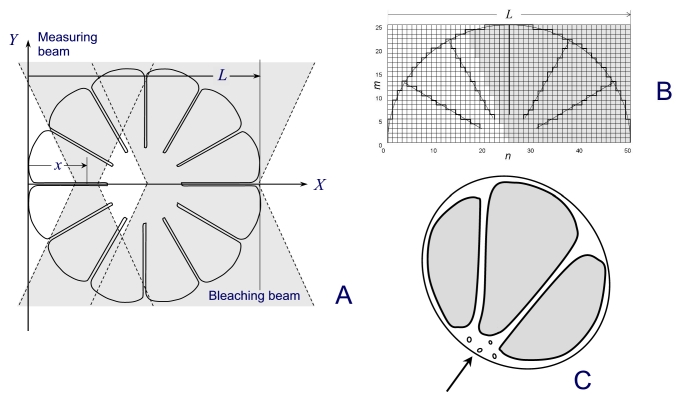
Geometry of ROS discs. **A** shows model of amphibian disc used for computing the effect of disc incisures on the rate of rhodopsin diffusion. Shaded wedge-shaped areas show geometry of bleaching and measuring beam in the microspectrophotometer. *L* denotes the ROS diameter, and *x* is the coordinate, as in Equation (2). **B** shows scheme of the grid for finite-difference solution of two-dimensional diffusion equation for the structure depicted in **A.** Shaded area is covered by the bleaching flash. Heavy lines delineate the borders at which the diffusion fluxes are set to 0. **C** shows schematic of the cross-section of Tokay gecko ROS based on our electron microscopy data. The arrow points to longitudinal cytoplasmic channel that started at the connecting cilium and ran along the entire length of the outer segment. Incisures radiated from the channel and separated the discs into a few isolated lobes.

Since the rod outer segment is not a rectangular slab, more realistic configurations should be considered. The least complication results from circular cross-section model and real geometry of measuring and bleaching beams in the microspectrophotometer. A finite angular aperture of the condenser (typically 0.4 to 0.5) means that the bleaching beam, even if perfectly focused, extends to the supposedly unbleached half of the outer segment. Similarly, absorbance measurements yield concentration averaged over a double-wedge shaped volume within the ROS rather than a point reading (Figure 10A). Both factors result in decreased efficient diffusion distance thus leading to overestimate of the diffusion constant. Yet the most serious problem is posed by the petal-like structure of amphibian ROS discs that may substantially hinder the diffusion. Poo & Cone [8] and Wey and Cone [15] suggested that the effect of incisures can be accounted for by introducing a correction factor *a* in Equation 1 as shown (Equation 3):

$$D=a\cdot D_{a}=a\frac{L^{2}}{\pi^{2}\cdot \tau }$$

By measuring heat dissipation in a disc model made of metal sheet, they found that *a* ≈2.7. To take into account both the geometry of bleaching/measuring beam and the petal-like structure of ROS discs, we made numerical calculations of diffusion in the structure outlined in Figure 10A,B. Due to the symmetry of the model with respect to *x*-axis, computations may only be performed on the upper half of the model. It was represented with a square lattice of *m* x n=25×50 cells, the cell’s size being Δ*x*=Δ*y*=*L*/50 (Figure 10B). Two-dimensional diffusion equation was solved with the simplest form of finite-difference method (Equation 8.49 in [30]), by a program written in Microsoft Visual Basic. The finite-difference equation was as follows (Equation 4)

$$\Delta c_{m,n}=\frac{D\cdot \Delta t}{\Delta x^{2}}\left[\left(c_{m,n-1}-2\cdot c_{m,n}+c_{m,n+1}\right)+\left(c_{m-1}-2\cdot c_{m,n}+c_{m+1,n}\right)\right]$$

Here Δ*c* is the concentration change, and Δ*t* is the time integration interval. At *t*=0, the cells in the shaded area of Figure 10B (“bleached” cells) were assigned *c_m,n_*=0 while the rest of the cells were assigned *c_m,n_*=1. During further computations, diffusion fluxes across the borders delineated with heavy lines were set to zero.

Qualitatively similar to the slab model, the equilibration followed a two-phase curve. Its initial part might be either shallow or steep, depending on the position *x* of the measuring beams within the ROS. It was followed by a single-exponential phase, whose slope allowed estimating the diffusion constant using Equation 3. The correcting factor *a* more critically depended on the depth of incisures than on their number. From the scarce transverse electron microscopic sections available in literature, we crudely estimated that incisures extend toward the center by 75% of discs' radius. This yielded *a* ≈2.8 for the correcting factor accounting for the combined effect of beam and disc geometry.

After correction, average diffusion constant obtained by pooling amphibian data of Table 1 is

*D*=(4.0±0.4)×10^−9^ cm^2^s^−1^. This is close to the generally accepted value of about 5×10^−9^ cm^2^s^−1^. The agreement with “classical” data are only apparent, though, because the correction factor *a* ≈2.7 has been applied inconsistently in earlier works. Thus, Poo and Cone [8] only applied it to frog, but not to *Necturus* rods. Instead, they used for *Necturus a*≈0.9 believing that the incisures in *Necturus* are very shallow. Liebman’s group [9-12] did not apply the correction at all. However, both species have an extensive system of deep incisures, as seen from electron microscopy (*Necturus* [12,31], frog [32,33]). When properly corrected for disc geometry, old data yield *D* ≈15×10^−9^ cm^2^s^−1^, roughly fourfold higher than obtained by us. Lower than classical diffusion constant, 3.2×10^−9^ cm^2^s^−1^, was also found in bleaching experiments on catfish cones [34]. Since no Meta III is formed in cones, and cone discs lack radial incisures, this value should represent “true” *D* rather than *D_a_*. Remarkably, the *D_a_* value from our absorbance measurements is in good agreement with earlier fluorescence photobleaching and recovery data on bullfrog rods [15] and recent results on *Xenopus* tadpoles [29] that are insensitive to Meta III formation. Both groups applied the same factor of 2.7 to account for the effect of incisures, and arrived at *D* of approximately 5×10^−9^ cm^2^s^−1^. Thus, the generally accepted value of *D* is probably correct, fortuitously resulting from mutual compensation of the two errors, experimental and computational.

### Immobile rhodopsin fraction is largely not due to fragmentation of ROS discs

We show that in all the species studied there exists a fraction of immobile rhodopsin molecules that can vary between essentially zero to 100% (Figure 4, Figure 5, Figure 6, and Figure 8; Table 1). The most obvious explanation for grossly incomplete equilibration of rhodopsin would be discontinuity of the disc membranes. If, for instance, some petals were pinched off, rhodopsin enclosed therein would not be able to mix with the pigment in the rest of the disc. The idea is apparently supported by electron microscopy data that show that some incisures can join near the disc's center, thus forming isolated lobes [12,31-33]. The effect of such arrangement on *D_a_* would depend on orientation of the lobes. If the incisures separated bleached and unbleached halves of ROS, they could completely stop the diffusion. The incisures running parallel to the direction of diffusion would have no effect. Drzymala et al. [12] found that in *Necturus* rods the immobile fraction could reach 15% and attributed it largely to this factor. In addition, they considered the possibility that a part of immobile fraction consists of rhodopsin molecules attached to incisures and the disc outer rim.

It is hard to quantitatively estimate the percentage of isolated lobes, and hence to assess their effect on the size of nondiffusible rhodopsin fraction. Yet our measurements of retinaloxime diffusion suggest that most discs are continuous structures without structural barriers for diffusion. Retinaloxime is a hydrophobic small molecule dissolved in lipid bilayer. Its “jumps” across cytoplasmic gaps seem unlikely. Nevertheless, it equilibrates quickly and completely within the ROSs that maintain a substantial rhodopsin gradient (Figure 7). Continuity of individual disc membranes in amphibian rods is also supported by free diffusion of retinol. Similarly to retinaloxime, retinol equilibrated quickly and completely in spite of limited diffusion of rhodopsin.

The idea of poor passability of incisures for retinaloxime is further supported by the results obtained in gecko. Electron microscopy of ROS sections made in the disc plane show that in gecko incisures indeed cut the discs in three or four completely separated lobes (schematic in Figure 10C). If the ROS were orientated in the MSP sample in such a way that the incisures run along the diffusion path, they would pose less hindrance to rhodopsin diffusion. Hence, movable rhodopsin would equilibrate faster than in amphibian rods (Figure 4, Figure 5, and Figure 9A), and it is expected that in gecko *D_a_ ≈D*. Indeed, gecko’s *D_a_*=4.2±0.3 (Table 1) is virtually identical to the amphibians’ *D*-value corrected for the effect of incisures. However, the incisures are virtually impassable for rhodopsin and poorly passable for retinaloxime. So if the incisures were oriented across the diffusion path, a big immobile rhodopsin fraction would be accompanied with greatly retarded oxime diffusion, as actually observed (Figure 9). Such reasoning led us to suggest that the immobile rhodopsin fraction observed in amphibian rods is largely not due to structural discontinuity of the disc membrane.

### Oligomeric rhodopsin arrays are a possible explanation

A possible explanation for large immobile fraction of rhodopsin molecules could be formation of big oligomeric arrays discovered recently with the Atomic Force Microscopy (AFM) [18-20]. AFM of mouse rod discs shows rhodopsin dimers often joined to form large paracrystalline areas on the membrane surface. Dimeric and oligomeric organization of rhodopsin in the disc membrane was further supported by conventional electron microscopy and various preparative biochemical techniques [35,36]. Such organization is supposed to increase the efficiency of interaction of photoactivated rhodopsin with transducin [37-44]. However, the existence of rhodopsin dimers, let alone higher oligomers, was doubted based on a previous data suggesting that rhodopsin in the native membrane is monomeric (see [45-47]). It is supposed that formation of rhodopsin dimers and paracrystalline areas can be related to nonnative conditions in AFM samples [45].

Our data on diffusion constant of “free” rhodopsin fraction do not allow discriminating between monomers and dimers. The theory that would connect the diffusion constant, either lateral or rotational, to the size of diffusing particle requires exact knowledge of membrane viscosity and possible interaction of rhodopsin with membrane lipids [17]. To date, no independent data on these factors are available. Therefore, the involved uncertainties preclude distinguishing monomeric and dimeric rhodopsins based solely on their rate of diffusion.

Extended paracrystalline areas, if they existed, would render rhodopsin virtually immobile within the time scale of our experiments. Thus the big immobile rhodopsin fraction found by us could result from formation of such oligomeric structures. The size of the fraction could vary greatly among cells, from virtually zero to complete lack of diffusion. This is in line with earlier findings. When measuring rotational diffusion of rhodopsin, Cone [16] noticed that in some retinal samples the relaxation of photo-induced dichroism occurs in two stages: a rapid initial decay followed by a larger and slower final decay. This may indicate the presence of higher rhodopsin oligomers. Coke et al. [48] traced rhodopsin rotation with a fluorescent probe. They detected substantial immobile fraction of rhodopsin whose size reversibly changes from nearly zero to 35% when varying temperature between 35 °C and 5 °C. Also, Wei et al. [15] reported that the degree of recovery of fluorescence after photobleaching appears to depend upon the condition of the animal and handling of the sample. Great variability of immobile rhodopsin fraction suggests that formation of the paracrystalline areas can be a dynamic process possibly regulated by some unknown factors.

What could be consequences of rhodopsin oligomerization for phototransduction? Proponents of oligomeric organization of rhodopsin suggest that the ordered structure somehow facilitates rhodopsin-transducin interaction [37-44]. For instance, stochastic simulation shows that ordered packing of immobile rhodopsins can increase the rate of activation of freely moving transducins [43]. The idea is apparently backed by experimental results [39] that demonstrate that the rate of transducin activation increases in detergents that favor formation of big rhodopsin oligomers. Measurements of transducin activation in this work, however, were conducted at complete rhodopsin bleach, and yielded maximum rate of 0.18 T s^−1^ per photoactivated rhodopsin. (This was estimated from Figure 2 in Jastrzebska et al. [39] by taking into account T/Rh molar ratio of about 8 in the sample). For comparison, the rate of transducin activation by a single rhodopsin in frog rod is ≈180 s^−1^, i.e., by three orders of magnitude higher [49]. Thus the results in vitro can hardly support speculations related to physiologic conditions.

It seems that within the traditional framework, rhodopsin oligomerization would virtually completely stop any signaling activity. It is firmly established both in biochemical and physiologic experiments that absorption of a single photon leads to activation of hundreds of transducins/PDEs per second, thus providing the first step of signal amplification. Therefore, multiple diffusion encounters between rhodopsin and transducin are prerequisite for any workable scheme of phototransduction. It should be noted that the mobility of rhodopsin as such is not crucial for the present scheme of cascade activation. The frequency of collisions between rhodopsins and transducins, hence the rate of transducin activation, is proportional to the *sum* of lateral diffusion constants of the two molecules (e.g., [1,50]). Therefore, movement of just one of the components would be sufficient to ensure multiple encounters and activations. The data of Bruckert, Chabre, and Vuong [51] suggest that transducin moves in the membrane plane faster than rhodopsin, so the rate of transducin activation is grossly independent of rhodopsin mobility.

The advantage of ordered packing of rhodopsins for transducin activation supposed by stochastic simulation [43] is deduced from comparison with randomly spaced *immobile* visual pigment. Allowing rhodopsins to move would probably abolish this advantage. Moreover, calculations in [43] rest on the assumption of free mobility of transducins within tightly packed paracrystalline rhodopsin domains. Yet formation of paracrystalline rhodopsin areas implies existence of a meshwork of interactions (of whatever sort) between neighbor molecules. Therefore it seems unlikely that multiple transducins, each connected by two anchors to the lipid bilayer, could move freely within the paracrystalline areas to meet a single photoactivated rhodopsin molecule inside the domain. A more plausible option is that most of the rhodopsins in the oligomeric arrays would be virtually excluded from phototransduction. Thus we suggest that rhodopsin oligomerization, if physiologically controlled, could be a mechanism of adaptation that would adjust photoreceptor sensitivity accordingly to its operating conditions.
